# The potential of P2X7 receptors as a therapeutic target, including inflammation and tumour progression

**DOI:** 10.1007/s11302-017-9593-0

**Published:** 2017-11-21

**Authors:** Geoffrey Burnstock, Gillian E. Knight

**Affiliations:** 1 Autonomic Neuroscience Centre, University College Medical School, Rowland Hill Street, London, NW3 2PF UK; 2 Department of Pharmacology and Therapeutics, The University of Melbourne, Melbourne, Australia; 3 Florey Institute of Neuroscience and Mental Health, Parkville, Melbourne, Australia

**Keywords:** Pain, Infection, Cancer, CNS disorders, Cardiovascular, Airways, Diabetes, Kidney, Bladder, Liver, Gut, Immune cells

## Abstract

Seven P2X ion channel nucleotide receptor subtypes have been cloned and characterised. P2X7 receptors (P2X7R) are unusual in that there are extra amino acids in the intracellular C terminus. Low concentrations of ATP open cation channels sometimes leading to cell proliferation, whereas high concentrations of ATP open large pores that release inflammatory cytokines and can lead to apoptotic cell death. Since many diseases involve inflammation and immune responses, and the P2X7R regulates inflammation, there has been recent interest in the pathophysiological roles of P2X7R and the potential of P2X7R antagonists to treat a variety of diseases. These include neurodegenerative diseases, psychiatric disorders, epilepsy and a number of diseases of peripheral organs, including the cardiovascular, airways, kidney, liver, bladder, skin and musculoskeletal. The potential of P2X7R drugs to treat tumour progression is discussed.

## Introduction

P2X receptors are a family of ionotropic ATP-gated receptors. They are cation-selective channels, equally permeable to Na^+^ and K^+^ and with significant Ca^2+^ permeability. Seven mammalian subunits have been cloned to date (P2X1-7) that form either functional homo- or heterotrimers. When three molecules of ATP bind to a P2X receptor, the pore opens within milliseconds, allowing the cations to flow. P2X7 receptors (P2X7R) differ in that they have extra amino acids in the intracellular C terminus and are bi-functional. The binding of ATP within milliseconds induces the opening of a channel selective for small cations, and within seconds, a larger pore opens, which allows permeation by molecules with a mass of up to 900 Da (leading to the release of inflammatory cytokines and apoptosis) (see [1, 2]).

An important review was published about the development of P2X7R antagonists for anti-inflammatory therapy, including therapeutic potential and recent discoveries [3]. More recent reviews about P2X ion channel receptors and inflammation [1] and the P2X7R as a therapeutic target [4] have been published. This review is focused largely on the recent interest since 2014 about the involvement of P2X7R in inflammation and tumour progression.

## P2X7R and inflammation

P2X7R have been implicated in the regulation of inflammation [5] and virtually all immune cell types (both of innate and adaptive immunity, including lymphocytes, macrophages, monocytes, neutrophils, basophils, dendritic cells, eosinophils and mast cells) express P2X7R [6]. They therefore represent significant therapeutic potential. A review was published in 2015 that discussed those P2X7R antagonists that exhibited promising therapeutic potential for the treatment of inflammatory diseases, pain and cancer [7]. Other reviews have been published that highlight the involvement of P2X7R in neuroinflammation and how medicinal chemists are working to identify centrally penetrant antagonists [8, 9]. For instance, GSK1482160 is a potent P2X7R antagonist with blood–brain barrier penetration, which has the ability to be radiolabelled with ^11^C. This allows it to be potentially useful as a biomarker of neuroinflammation [10], since P2X7R may mediate the neuroinflammation and cognitive impairment that can result following surgery [11]. Another review explored the role of P2X7R in fibrosis, the pathological outcome of most chronic inflammatory diseases [12].

**Fig. 1:**
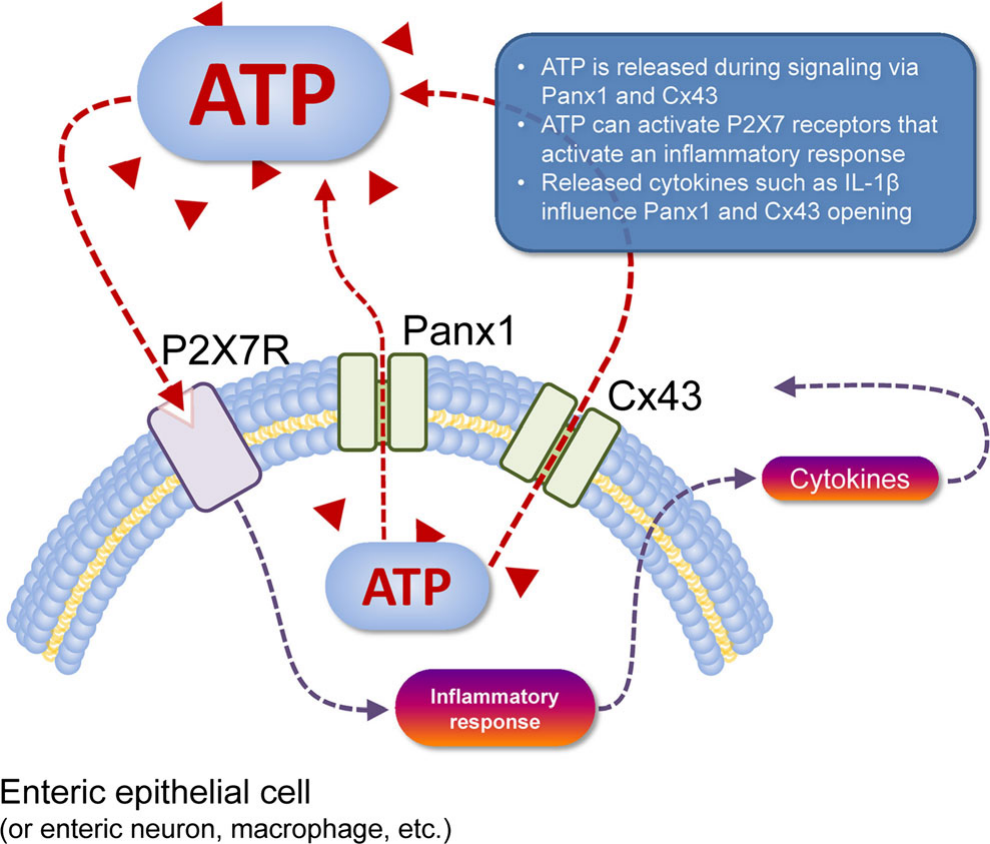
Cell type-specific schema of ATP release and action. ATP (red triangles) can be released from the cell cytosol to the extracellular space (dashed red line) via pannexin 1 (Panx1) channels or connexin 43 (Cx43) hemichannels (pictured). Once in the extracellular space, this ATP acts as a paracrine transmitter, as can ATP released from nearby cells that are dead or dying (not shown). Extracellular ATP can activate P2 receptors, such as P2X7 receptors (P2X7R) (pictured), that depolarise the target cell but also activate an inflammatory response in immune cells (dashed gray line) with subsequent release of cytokines such as interleukin (IL)-1β that can act back at Panx1 and Cx43 to modulate their function (dashed gray lines). Activation of P2X7R also mediates the T cell responses (e.g. Ca^2+^ entry, IL-2 synthesis) and macrophage migration (not shown). (Reproduced from [15], with permission from Frontiers Media S.A. (via Open Access))

Activation of P2X7R is associated with immune responses, including allergic inflammation [13]. ATP released from damaged or infected cells causes inflammation by activation of P2X7R and subsequent release of inflammatory cytokines, such as interleukin (IL)-1β, thereby acting as a danger signal by activating P2X7R on immune cells to increase immune responses [14]. An example of this is seen in enteric epithelial cells in conditions of inflammation of the gut (see [1]; Fig. 1). Pulmonary neutrophils are the inflammatory cells that are initially recruited during lung injury. Antagonism of the P2X7R with AZ106006120, or knockout (KO) of the receptor, reduced neutrophil infiltration and pro-inflammatory cytokine levels in a mouse model of acute lung injury [16]. Human and murine neutrophils express functional P2X7R, which mediate NLRP3 inflammasome-dependent IL-1β secretion [17]. Nanobodies are small, single-domain antibody fragments and a mouse nanobody, 13A7, was shown to block gating of the P2X7R channel on T cells and macrophages in vivo and is proposed as a new drug candidate for inflammatory disorders [18].

## P2X7R in inflammation, infection and immunity

Extracellular ATP is an endogenous danger signal that activates inflammatory responses in immune cells. The role of P2X7R in infectious inflammatory diseases has been reviewed [8, 19, 20]. P2X7R are needed for the development of the inflammatory response associated with sepsis in mice [21, 22] and Brilliant Blue G (BBG; a selective antagonist for mouse P2X7R that is blood–brain barrier permeable and safe) was shown to ameliorate sepsis-induced brain damage [23]. Also in mice, P2X7R downstream of caspase-11 play a critical role for pyroptosis and susceptibility to sepsis induced by the non-canonical inflammasome [24].

The use of P2X7R agonists in conjunction with low molecular weight anti-bacterial medicines has been proposed for the treatment of multi-drug-resistant tuberculosis [25]. P2X7R antagonists are potential tools for the treatment of *Clostridium perfringens* type C [26] and *Porphyromonas gingivalis* [27] infections. P2X7 activation was shown to be protective during severe *Escherichia coli* infection in mice [28]. Similarly, ATP release from infected macrophages and subsequent activation of P2X7R are critical for IL-1-dependent host protection from *Bacillus anthracis* [29]. The role of P2X7R and ectonucleotidases in infectious inflammatory diseases has been reviewed [20].

Evidence was presented to show that mouse P2X7R are involved in containing the parasitic protozoan *Toxoplasma gondii* spread in vivo, by stimulating inflammation [30]. The dysfunction of P2X7R is likely to contribute to morbidity due to human schistosomiasis, a chronic inflammatory disease [31]. P2X7R-deficient mice were more susceptible to *Leishmania amazonensis* infection than wild type (WT) mice, suggesting that P2X7R play a key role in parasite control by regulating T effector cells and inflammation [32].

P2X7R activation regulates inflammatory responses during acute viral infection in mice [33]. Upregulated expression of P2X7R on peripheral blood mononuclear cells provides anti-viral immunity in patients against hepatitis C virus [34], while activation of P2X7R participates in the exacerbated immune response that occurs during influenza virus infection in mice [35]. P2X7R play a role in control of dengue virus-2 infection and KN62 appeared to have anti-viral and anti-inflammatory actions in infected human monocytes [36]. Purinergic receptors, particularly P2X7, have been identified as key mediators of human immunodeficiency virus-1 (HIV-1) infection and inflammation [37] and ATP induces rapid release of HIV-1 from virus containing compartments of human macrophages [38]. In HIV-1 infection, abnormalities in neuron-glia interactions result in neuronal damage. HIV-1 Tat induces neuronal apoptosis and augments the expression of P2X7R in astrocytes. Oxidised ATP (oxATP), A-438079 and BBG attenuated Tat-mediated neurotoxicity and may be novel tools for therapeutic management of neuroAIDS [39].

A valuable review discusses the role of purinergic signalling in autoimmunity and highlights a role for P2X7R in systemic lupus erythematosus [40]. P2X7R activation deleted intestinal T cells by apoptosis and suppressed T cell-induced colitis in mice [41]. P2X7R are expressed on peripheral lymphocytes and may influence the immune profile from patients with the indeterminate form of Chagas disease [42].

## P2X7R and inflammatory neuropathic pain

The earlier literature about the involvement of P2X7R in neuropathic pain has been reviewed [43].

Genetically determined P2X7R pore formation was shown to regulate variability in chronic pain sensitivity [44]. P2X7R activation by endogenous ATP contributes to the development of inflammatory hyperalgesia. Removal of the P2X7R gene or P2X7R antagonists, such as BBG and oxATP, abolished chronic inflammatory and neuropathic pain in animal models (see review by Alves et al. [45]). Inflammatory pain that occurs in burn patients following dressing changes was relieved by puerarin treatment, which was claimed to act by decreasing the expression levels of P2X7R mRNA and protein in peripheral blood mononuclear cells [46]. P2X7R activation in vivo was involved in the development of central sensitisation in an acute inflammatory pain animal model (see [1]).

P2X7R antagonists were shown to have analgesic activity in a rat model of neuropathic pain. P2X7R expressed by microglia mediate neuropathic pain, which is reduced by antagonists, such as A-740003 and A-438079 (see [47]). Central nervous system (CNS)-penetrant P2X7R antagonists may be beneficial for the treatment of persistent pain by targeting microglia. Resveratrol was shown to be neuroprotective against neuropathic pain mediated by P2X7R expressed on satellite glial cells of the dorsal root ganglia (DRG) [48].

## P2X7R and tumour progression

A review covering the early literature about the involvement of purinergic signalling and cancer, including discussion about the roles of the P2X7R in a variety of tumours, is available [49].

**Fig. 2:**
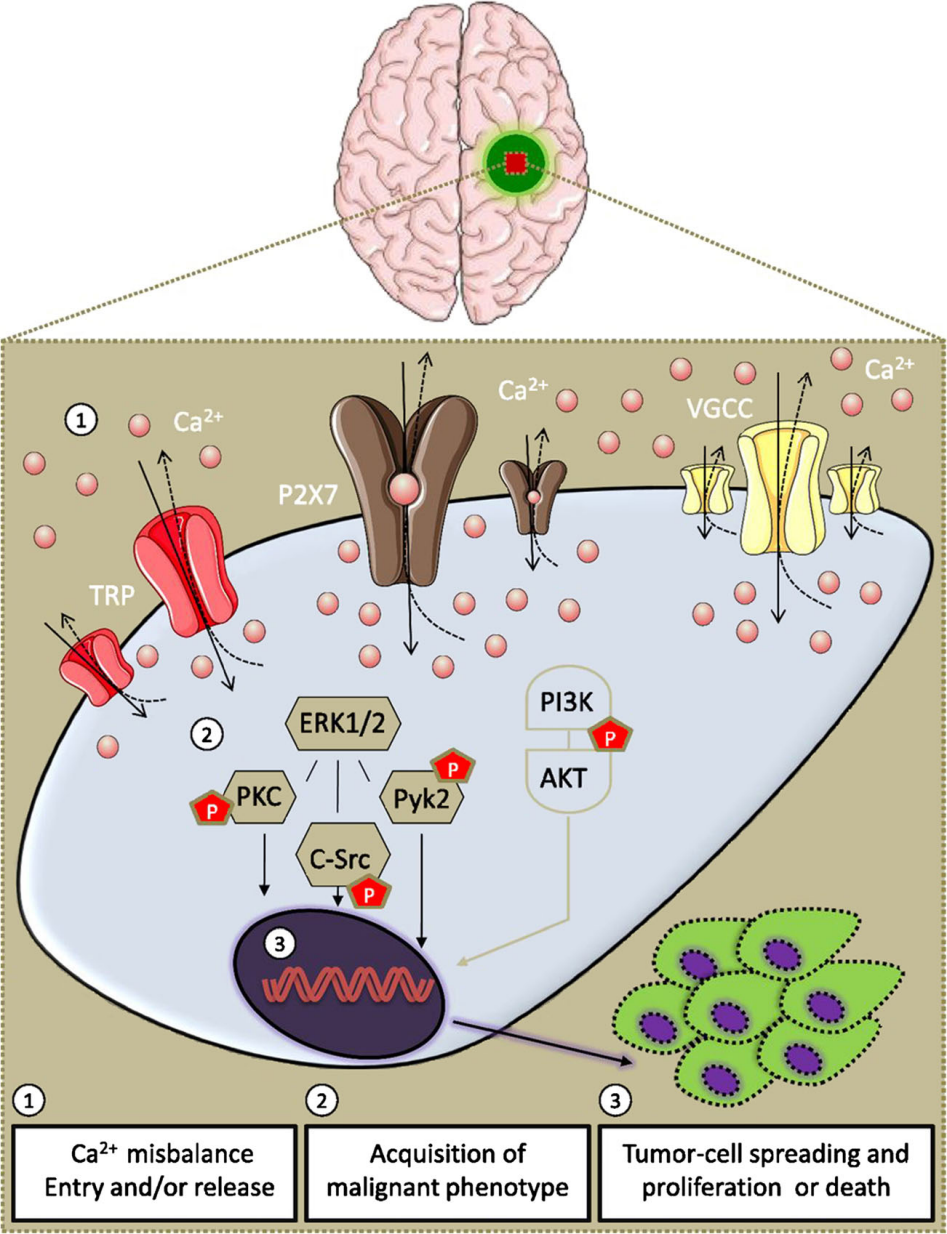
Altered regulation of calcium channels in brain tumours is part of neoplastic transformation. In the brain, the transformation of a normal cell into a tumour cell might be related to Ca^2+^ oscillations, and the homeostasis misbalance can define the malignant phenotype, which includes uncontrolled proliferation, enhanced migration and invasion and abnormal cell death. The activation of P2X7 receptors (P2X7R) leads to extracellular signal-regulated protein kinases 1 and 2 (ERK1/2), phosphatidylinositol 3-kinase (PI3K) and mitogen-activated protein kinase 1/2 (MEK1/2) activation. High P2X7R functionality and pore activity are linked to apoptosis/necrosis in glioma cells and better progression-free survival. (Reproduced from [55], with permission from the American Society for Pharmacology and Experimental Therapeutics)

P2X7R are a key mediator of inflammation and cancer invasion/metastasis and P2X7R antagonists are potential anti-metastatic agents [50]. A feature of some cancer cells is the high level of expression of P2X7R, which, depending on the tumour type, can mediate either proliferation or cell death (see [51]). Thus, P2X7R activation may have effects on anti-tumour immunity that are opposed to a direct effect on tumour growth. It was also pointed out in this review that there is increased release of ATP, promoting cancer cell migration and metastasis. High levels of extracellular ATP accumulate in tumour interstitium and high ATP doses inhibit migration of endothelial cells from human breast carcinoma, via the activation of P2X7R [52]. Human pancreatic duct adenocarcinoma cells in vitro express high levels of P2X7R protein and AZ10606120 inhibited cell proliferation [53]. ATP activation of P2X7R triggers immunogenic signalling, which converts dying cancer cells into an effective anti-cancer vaccine [54]. P2X7R activation is linked to elevated expression of inflammation promoting factors, tumour cell migration, increase in [Ca^2+^]_i_ and membrane depolarisation in malignant gliomas (see [55]; Fig. 2). Evidence has been presented to suggest that P2X7R antagonists are promising therapeutic tools for the treatment of osteosarcoma [56]. Growth of experimental tumours is strongly inhibited by P2X7R antagonism of both cancer and immune cells (see [57]). A non-functional P2X7R, nfP2X7R, is expressed on cancer cells and has been proposed as a novel therapeutic target for human cancer [58, 59]. A phase I clinical trial has recently demonstrated that nfP2X7R-targeted antibodies were a novel, safe and tolerable topical therapy for basal cell carcinoma [60]. P2X7R activation induces apoptosis in acute myeloid leukaemia cells but not in normal hematopoietic stem cells [61].

ACN human neuroblastoma cells express P2X7R, which is a regulator of its growth and angiogenesis, and AZ10606120 and A-740003 reduced ACN-derived tumour growth in nude mice so P2X7R may be a novel therapeutic target for treatment of neuroblastoma [62, 63]. P2X7R are expressed on human malignant glioma and C6 cells [55, 64, 65] and high P2X7R expression has been correlated with progression-free survival and overall survival [66]. P2X7R activation caused release of inflammatory cytokines by macrophages exposed to glioma-conditioned medium that was prevented by A-740003 [67]. A P2X7R agonist, 2'(3')-*O*-(4-benzoylbenzoyl)-ATP (Bz-ATP), enhanced the temozolomide anti-tumour effect on human cultured glioblastoma stem cells [68].

Autocrine release of ATP and activation of P2X7R influence the metastatic migration of human lung cancer H292 and PC-9 cells [69], and in immunodeficient mice, migration of transplanted HTB177 and HTB183 lung cancer cells was reduced by A-438079 [70]. The role of P2X7R in tumour progression is complex and studies show contradictory effects of P2X7R activation. For instance, an increase in survival of non-small cell lung cancer patients with high P2X7R expression was observed [71], although low P2X7R expression resulted in greater mRNA (miR-21) expression in the non-small cell lung cancer tumours. Thus, the high levels of miR-21 expression in these cancer patients may be a consequence of P2X7R downregulation and resultant promotion of tumour progression. These results agree with the study by Souza and colleagues [72], which showed that defective P2X7R expression following miR-21 activation by a K-Ras mutation, led to reduced tumour-killing activity, resulting in a poorer prognosis. P2X7R are expressed in pancreatic cancer PancTu-1 Luc cells and antagonists, such as AZ10606120, are likely to be effective therapeutic agents [53]. Other antagonists, A-438079 and A-740003, reduced inflammation associated with colitis but increased tumour incidence in a mouse model of colitis-associated cancer [73]. In P2X7R-transfected human embryonic kidney cells and CT26 colon carcinoma, there was enhanced tumourigenesis when the cells were inoculated into either immunodeficient or immunocompetent mice, respectively. It was shown that in tumours derived from B16 mouse melanoma or ACN human neuroblastoma cell lines, tumour growth was inhibited by oxATP [74]. The anthraquinone, emodin, suppressed the invasiveness of the highly invasive breast cancer cell line MDA-MB-435s, by antagonizing P2X7R [75]. ATP increased [Ca^2+^]_i_ in breast tumour cells and high concentrations produced apoptosis via P2X7R. ATP-mediated activation of the human breast cancer cell line T47D resulted in an increase in cell migration and the development of metastases, suggesting a potential therapeutic role for P2X7R antagonists [76]. P2X7R expression is a prognostic indicator for postoperative cancer-specific survival of patients with clear-cell renal cell carcinoma [77].

Decreased expression of P2X7R is associated with the development of cervical cancer. In women, decreased expression of P2X7R is found in endometrial epithelial pre-cancerous lesions. Activation of P2X7R-dependent apoptosis with BzATP may be a chemotherapeutic approach to prevent cell growth of pre-cancerous and early cancerous epithelial lesions [78]. Clearly, while ATP and P2X7R activation induced apoptosis of tumour cells in some models, in others it accelerated tumour growth.

It is known that there are many polymorphisms of the P2X7R [79, 80], which, in addition to resulting in a loss of function, may alter the activity of the receptor. Expression of non-functional cytolytic P2X7R was found in all pathology specimens of prostate cancer examined [81]. P2X7R were not expressed in normal tissues from patients with no evidence of cancer, suggesting that the appearance of P2X7R is an early marker of prostate cancer. The expression of P2X7R is increased by hypoxia and hypoxia-driven increase in P2X7R enhances invasion and migration of tumour cells [82]. Ultraviolet (UV) light has been implicated in the genesis of cutaneous cancer, including basal and squamous cell carcinoma as well as melanoma. UV-B irradiation destroys P2X7R, by directly killing epithelial cells and by reducing P2X7R mRNA by degrading the protein, and may contribute to the malignant transformation of keratinocytes [83]. P2X7R were expressed in the necrotic centre of nodular basal cell carcinomas and in apoptotic cells in superficial multifocal and infiltrative cells. ATP caused apoptosis of cultured A431 human squamous cell carcinoma cell line via P2X7R and application of BzATP inhibited the formation of skin papillomas and carcinomas in mice [84]. There was increased expression of P2X7R in patients with superficial spreading melanomas, which were later shown to be functional, and it was suggested that they are a target for melanoma therapy. A low pH environment (like that seen in solid tumours) induced ATP release from B16 melanoma cells to increase proliferation via P2X7R and oxATP inhibited tumour growth in B16 melanoma-bearing mice [85]. In P2X7R KO mice, tumour progression of B16 melanoma was accelerated, showing that P2X7R are critical to support an anti-tumour immune response as well as restricting tumour growth and metastatic diffusion [86]. γ-Irradiation, which causes growth arrest and death of tumour cells, induced P2X7R-dependent ATP release from the B16 melanoma cells. P2X7R KO mice were susceptible to bone cancer pain and had an earlier onset of pain-related behaviours. The majority of human osteosarcomas express P2X7R isoforms A and B and the expression of either isoform is differently coupled to cell growth and activity [87]. P2X7R are involved in cancer-induced bone pain and A839977 was suggested as a useful analgesic tool in a rat model of cancer-induced bone pain [88].

Activation of P2X7R can either promote cellular survival or induce cytotoxicity and depends on the stimulus intensity of ATP to control the ion channel or the P2X7-dependent large pore functions. How these two opposite effects are controlled is not fully understood. Recently, a feedback loop was described showing that sustained activation of P2X7R results in the release of active matrix metalloproteinase 2, which stops ion channel and large pore responses in several different cell types, including macrophages and human tumour cells. This effect may be an important fine-tuning of P2X7R functions. The authors suggested that P2X7R antagonists could be useful as in treating inflammatory diseases and cancers [89].

As the tumour microenvironment contains very high concentrations of ATP, adenosine is also present in high concentrations, following enzymatic breakdown of ATP by CD39 and CD73. Both ATP and adenosine contribute to immunosuppression or immunostimulation of the host, and stimulation of growth or cytotoxicity of the tumour, depending on the receptors activated. Thus, targeting specific receptor subtypes produces different effects, for example targeting CD73 or A_2A_ receptors reduced immunosuppresion and potently inhibited tumour growth [90]. As has been described above, growth of experimental tumours is also strongly inhibited by targeting P2X7R of cancer and immune cells (see [57]).

## P2X7R and diseases of the CNS

**Fig. 3:**
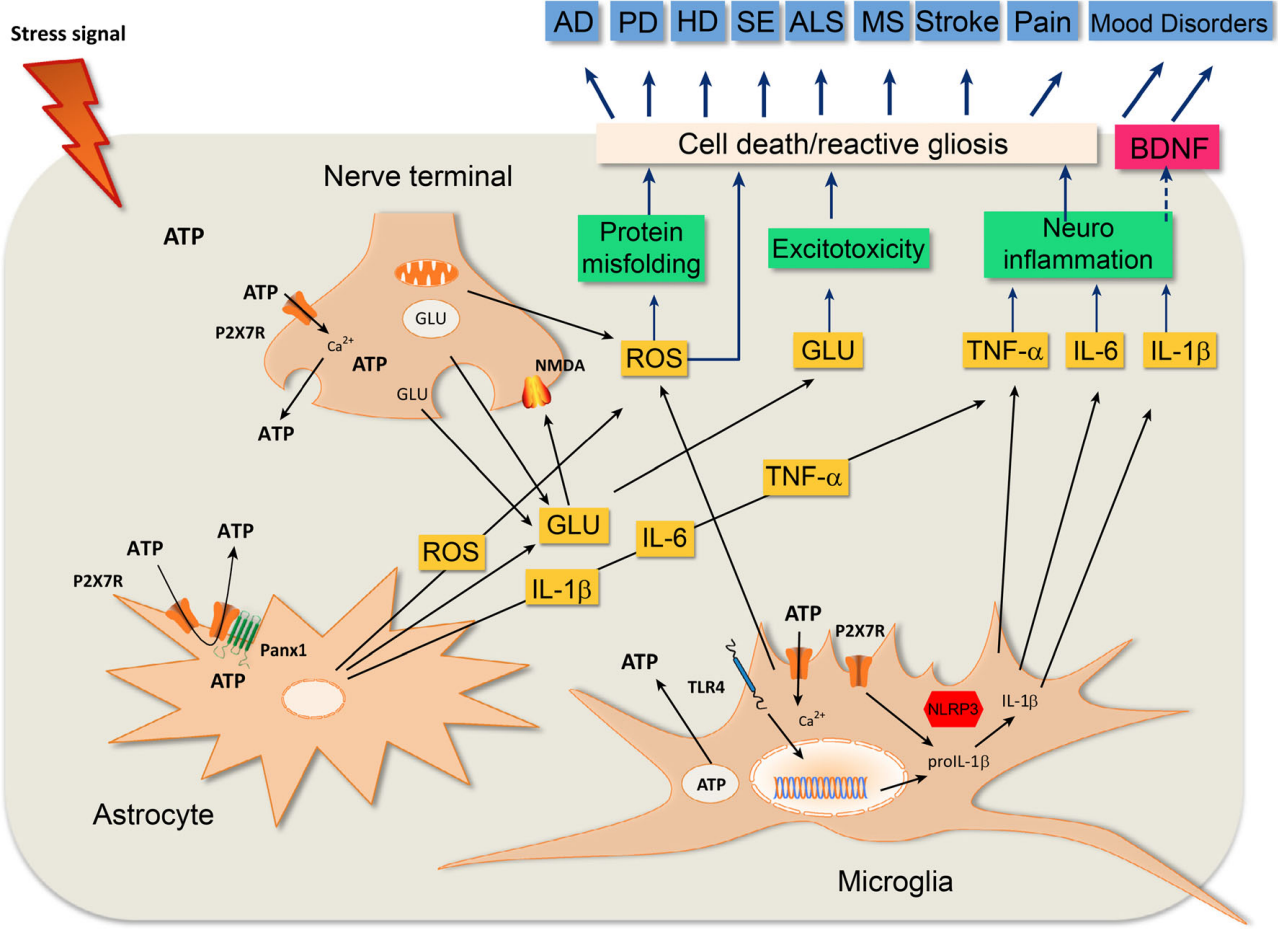
Common disease mechanism by P2X7 receptor (P2X7R)-mediated pathways in central nervous system (CNS) disorders of different etiology. P2X7R are expressed on nerve terminals, astrocytes and microglia, and they are upregulated in various disease conditions. Stress signals such as hypoxia/ischemia (metabolic limitations), mechanical injury and bacterial or chemical toxins elicit the endogenous activation of P2X7R and lead to a self-amplifying ATP release and to further activation of P2X7R on neighbouring cells. Following the influx of Ca^2+^ through the receptor ion channel complex, P2X7R activation (i) releases glutamate from nerve terminals and astrocytes by both exocytotic and non-exocytotic mechanisms, which may give rise excitotoxicity; (ii) leads to the posttranslational processing of pro-interleukin-1β (pro-IL-1β) to the leaderless, mature IL-1β and to its further release by the NLRP3 inflammasome and that of other cytokines, which contribute to neuroinflammation; (iii) enhance reactive oxygen species (ROS) production and thereby aggravate protein misfolding and neuronal damage; (iv) leads directly or indirectly to cell death and the following reactive astrogliosis; and (v) directly or indirectly downregulates the production of brain-derived neurotrophic factor (BDNF) and the subsequent neuroplasticity. These key mechanisms could be manifested and contribute to disease pathology in Alzheimer’s disease (AD), Parkinson’s disease (PD), Huntington’s disease (HD), status epilepticus (SE), amyotrophic lateral sclerosis (ALS), multiple sclerosis (MS), stroke, pain and mood disorders in different forms and proportion, depending on the etiology. Abbreviations: GLU, glutamate, ROS, reactive oxygen species. (Reproduced from [92], with permission from Elsevier)

The role of P2X7R in diseases related to neuroinflammation and the use of centrally penetrant antagonists has been highlighted [1, 11, 14, 18, 91–94]. Blockade of P2X7R may serve as a therapeutic target in alleviating the degree of inflammation seen in neurodegenerative and neoplastic conditions [95]. Figure 3 is a schematic showing P2X7R-mediated pathways of common disease mechanisms in CNS disorders of different aetiology. P2X7R are upregulated in various disease conditions and stress signals elicit activation, which can lead to excitotoxicity, neuroinflammation, neuronal damage, reactive astrogliosis or neuroplasticity, contributing to disease pathology (see [92]).

### Neurodegenerative diseases

Attention has been directed recently towards the use of P2X7R antagonists for the treatment of neurodegenerative diseases, which are often associated with inflammation and damage to both neurons and glia [96–99]. P2X7R antagonists were neuroprotective in an animal model of Alzheimer’s disease (AD). P2X7R trigger α-secretase-dependent processing of the amyloid precursor protein to generate β-amyloid peptides, which are present in the amyloid plaques in AD. Inhibition of P2X7R in vivo reduces amyloid plaques through glycogen synthase kinase 3β and secretases. BBG improved cognition in an animal model of AD and inhibited amyloid-β-induced loss of filopodia and dendrite spines in cultured hippocampal neurons [100]. The control of amyloid plaque formation in vivo by P2X7R was reviewed in 2015 [97] and discussed further in 2017 [101]. It was suggested that P2X7R contribute to Parkinson’s disease (PD) pathogenesis through a triple effect on synaptotoxicity, gliosis and neurotoxicity, indicating therapeutic potential of P2X7R antagonists for PD [102–104]. BBG was neuroprotective in an intranigral lipopolysaccharide (LPS) animal model of PD [104]. P2X7R are involved in multiple sclerosis (MS) and in the animal model of MS, experimental autoimmune encephalomyelitis (see [105]), where increased expression of P2X7R protein was shown in brain homogenates. Genetic variants in P2X7R affect susceptibility to MS [106, 107]. Altered P2X7R level and function in mouse models of Huntington’s disease was reported and BBG prevented neuronal apoptosis and reduced body weight loss and motor-coordination deficits [108].

Inflammation features in the pathogenesis of amyotrophic lateral sclerosis (ALS). The potential of antagonism with BBG for the treatment of ALS was proposed, but it was claimed that the treatment was gender-dependent, although this varied in different studies using the SOD1-G93A mouse model of ALS: more effective in males [109], more effective in females [110] and no difference between the sexes [111]. Similarly, the pathogenesis of ALS was shown to differ in different studies. In P2X7R KO-SOD1-G93A mice, onset of the disease occurred more rapidly than in WT mice, with increased astrogliosis, microgliosis, motorneuron loss, induction of pro-inflammatory markers and activation of mitogen-activated protein kinase (MAPK) pathways [112]. In a later study by this group using the same mouse model of ALS, BBG reduced neuroinflammation at late pre-symptomatic phases of the disease, enhanced motor neuron survival and reduced microgliosis in the lumbar spinal cord [111]. These differences may be due to the timing of the receptor block, complete absence of the receptor in the KO study, as opposed to antagonising the receptor prior to symptoms showing in the latter study. Ca^2+^ abnormalities in ALS exist in both motor neurons and immune cells. Reduction of P2X7R expression on peripheral blood mononuclear cells from ALS patients led to calcium dysregulation, a feature of ALS [112].

### Psychiatric disorders

Activation of microglial P2X7R causes inflammation and brain penetrant P2X7R antagonists are being developed since microglial P2X7R are being increasingly recognised as a therapeutic target for the treatment of neurological and psychiatric diseases [113]. A review on this subject is available [114].

There is increased neuronal death via P2X7R and pannexin 1 channels in cultured cerebral cortex from prenatal LPS-exposed mice, which suggested a link to an increased risk of developing neurological disorders, including schizophrenia, in the offspring (see [115]). P2X7R, mediating neuroinflammation via the activity of microglia, may play a role in bipolar disorder and offer therapeutic possibilities (see [116]). Anti-depressant effects of chrysophanol (a natural anthraquinone) via inhibition of P2X7R has been reported in an LPS animal model of depression [117]. Similarly, BBG had anti-inflammatory and anti-depressant effects in mice after LPS administration [118]. Acute restraint stress activates the inflammasome via release of ATP and stimulation of P2X7R in a mouse model of stress-related mood disorders [119] and the antagonist A-804598 was shown to have an impact on the neuroimmune and behavioural consequences of stress in a rat model [120]. The P2X7R-Gln460Arg variant has been associated with mood disorders and co-expression of WT P2X7R with the polymorphic variant Gln460Arg transfected into HEK293 human cells was found to impair receptor function [121].

### Neuroprotection from injury

There are reviews that discuss the importance of inflammation and the involvement of purinergic signalling in the responses to brain injury [8] and of the P2X7R in particular [1, 14]. P2X7R antagonists are a promising therapeutic tool for the treatment of cerebral ischaemia (see [122, 123]). BBG reduced delayed neuronal cell death in the hippocampal CA1 region after ischaemia/reperfusion injury and it was found that systemic administration of P2X7R antagonists improved recovery after spinal cord injury. The neuroprotective action of the P2X7R antagonist, GSK1370319A, acts by inhibiting the assembly of the NLRP3 inflammasome in glial cells [124, 125]. ATP enhanced radiation-induced brain injury through microglial activation via P2X7R-mediated release of inflammatory mediators, such as cyclooxygenase-2, tumour necrosis factor-α (TNF-α) and IL-6, and P2X7R antagonists were suggested as a potential strategy for the treatment of patients with radiation-induced brain injury [126]. Activation of P2X7R in the hippocampus following traumatic brain injury was thought to imply cognitive impairment in rats [127], while A-438079 provided neuroprotection toward neurological disorders, including stroke, traumatic brain injury and subarachnoid haemorrhage, as well as preserving blood–brain barrier integrity [128].

Recent papers have recognised a role for P2X7R antagonists for the treatment of epilepsy, including drug-resistant epilepsy [8, 129, 130]. P2X7R antagonism produced lasting reduction in the development of spontaneous seizures and inhibited glial inflammatory responses in mouse models of experimental temporal lobe epilepsy [131] and pentylenetetrazol-induced seizures [132]. However, in pilocarpine-induced status epilepticus in mice, blockade of central P2X7R increased the number of seizures and their severity [133]. P2X7R are considered as a target for the treatment of hypoxic/ischaemic encephalopathy and other causes of neonatal seizures since in A-438079-treated mice, seizure number, EEG power and spiking during hypoxia as well as molecular markers of inflammation and microglia were reduced [130].

## Cardiovascular diseases

The long non-coding RNA (lncRNA), NONRATT021972, targeted using a small interfering RNA (siRNA), decreased the upregulation of P2X7R in the superior cervical ganglion and improved cardiac function after myocardial ischaemia in rats [134]. Myocardial ischaemic injury facilitated sympathoexcitatory action via P2X7R and it was suggested that P2X7R antagonists may be useful for the treatment of coronary heart disease as expression of P2X7R is increased during coronary ischaemia-reperfusion [135]. Microglial P2X7R in the rat hypothalamic paraventricular nuclei regulate the sympathoexcitatory responses in acute myocardial infarction and gene knockdown of P2X7R with P2X7-siRNA, or inhibition with BBG, reduced mRNA and protein expression of IL-1β and TNF-α [136]. P2X7R involvement in dilated cardiomyopathy has been reported in P2X7R KO mice [137]. A-740003 reduced experimental autoimmune myocarditis in a mouse model, suggesting a treatment for clinical myocarditis [138]. P2X7R in the kidney play a role in hypertension and it has been suggested that P2X7R antagonists may have promise as clinical anti-hypertensive agents. In atherosclerotic mice, P2X7R are over-expressed whereas P2X7R KO mice were found to have less plaque formation and decreased leukocyte recruitment following ATP stimulation [139]. P2X7R are pro-thrombotic and genetic KO of the P2X7R gene is protective in a mouse model of carotid artery thrombosis. Vasomotor dysfunction was caused by sub-failure overstretch injury in rat abdominal aorta via activation of P2X7R and this suggested that the use of antagonists for the treatment of vascular stretch injury in humans may be beneficial [140].

## Diseases of the airways

Inflammation occurs in most diseases of the airways, including asthma, chronic obstructive pulmonary disease, cystic fibrosis, dyspnea, allergy, infection and injury. P2X7R are a target for therapeutic intervention in lung hypersensitivity reactions associated with chronic inflammatory responses. Extracellular ATP was recognised as a danger signal activating P2X7R in lung inflammation and fibrosis and P2X7R antagonists were proposed as a novel therapeutic approach in humans to control IL-1β production and fibrosis in lung injury [141] as well as in silicosis, an occupational lung disease, following a study in a mouse model [142]. Attenuated P2X7R function gives protection from asthma and is age-related, being most effective in young boys. P2X7R have also been implicated in the pathophysiology of allergy-induced lung inflammation. Targeting P2X7R on haematopoietic cells, namely dendritic cells or eosinophils, may be a therapeutic approach for the treatment of allergic asthma. The anti-allergic anti-histamine, oxatomide, is claimed to also act as a P2X7R antagonist in N18TG2 and J774 cells [13]. BBG prevented neurogenic pulmonary oedema after subarachnoid haemorrhage in rats by attenuating inflammation [143]. Cigarette smoke activates P2X7R signalling, which appears to be involved in the pathogenesis of emphysema, and induces ATP and inflammatory cytokine release from neutrophils via P2X7R activation [144]. Polymorphisms of the P2X7R gene are associated with the risk and prognosis of human tuberculosis [145]. Data was presented to support the view that P2X7R antagonists should be used to treat the aggressive forms of tuberculosis [146]. ATP, released by activated macrophages and damaged cells, modulates lung inflammation in pneumonia in cattle. Both epithelial cells and pulmonary microvascular endothelial cells expressed mRNA for P2X7R, as did alveolar macrophages, which, when stimulated, activate the proinflammatory IL-1 to IL-5 cytokine cascade. P2X7R are involved in the pathophysiology of LPS-induced lung injury [147] and there is upregulation of pulmonary P2X7R in both acute and chronic lung injury in mice where P2X7R deletion was shown to be lung protective [148]. Pulmonary neutrophils are the initial inflammatory cells to be recruited during lung injury and are crucial for innate immunity. In contrast, pathological recruitment of neutrophils results in lung injury and P2X7R antagonists were shown to reduce neutrophil infiltration and proinflammatory cytokine levels [16].

## Gut disorders

Extracellular nucleotides and their receptors are involved in the pathogenesis of inflammatory bowel disease (IBD), which includes two main forms, namely ulcerative colitis (UC) and Crohn’s disease (CD). P2X7R are involved in colonic motor dysfunction associated with bowel inflammation in rats [149] and are over-expressed in gut mucosa of patients with CD [150]. P2X7R KO mice were protected against gut inflammation, while in WT mice, ATP via P2X7R triggers the death of mucosal regulatory T cells [151]. Oestrogen receptor β activation may play a therapeutic role in IBD by downregulation of P2X7R [152]. Reviews discussing the role of P2X7R in IBD are available [15, 153]. UC differentially affects P2X7R-expressing enteric neurons based on their chemical codes [154]. In a later paper, it was shown that trinitrobenzene sulfonic acid-induced UC in rats affected secretory and vasodilatory neurons, enteric sensory neurons and enteric glia of the submucosal plexus expressing P2X7R [155]. ATP mediates inflammatory responses in dextran sulfate sodium-induced UC in mice via P2X7R signalling and A-438079 down-regulated the production of proinflammatory cytokines and attenuated the colitis [156]. There is increased expression of P2X7R in the inflamed mucosa in CD in humans and animal models, suggesting that P2X7R may be a target for treatment of CD [151, 156, 157]. Purinergic signalling is involved in gastrointestinal motility disorders, such as diarrhoea and constipation. P2X7R activity was enhanced in enteric glia that were isolated from mice with long-term morphine treatment, which can result in colonic inflammation [158].

## Diseases of the kidneys

Increased expression of P2X7R in renal hypertension, polycystic kidney disease (PKD) and glomerulonephritis is opening up novel purinergic possibilities for the treatment of kidney failure (see [159, 160]). A-438079 protected against ischaemic acute kidney injury in mice [161]. P2X7R are expressed in collecting duct cysts in the *cpk/cpk* mouse model of congenital PKD, and mRNA and protein increased as the disease developed. ATP inhibits renal cyst growth, via P2X7R and oxATP, and A-438079 reduced cyst formation via MAPK-dependent pathways in a zebrafish model of PKD [162]. P2X7R expression has been demonstrated in both experimental and human glomerulonephritis, which suggests that P2X7R antagonists may have therapeutic potential [163]. P2X7R deficiency attenuated renal injury in experimental glomerulonephritis in mice. BBG attenuated nephritis by inhibiting inflammasome activation in a mouse model of lupus nephritis [164]. There is predominant P2X7R control of glomerular haemodynamics in angiotensin-II hypertension [165]. While P2X7R are only weakly expressed in healthy kidney, they are significantly upregulated in hypertension [166], antagonism of which would reduce interstitial inflammation, prevent interstitial cell death and improve blood pressure control. P2X7R antagonism prevented the development of salt-sensitive hypertension and renal injury in Dahl salt-sensitive rats [167]. P2X7R expression was increased in human kidneys with type 2 diabetes and activation of P2X7R evoked renal inflammation and injury in the high-fat diet model of metabolic syndrome, suggesting that P2X7R antagonists might be useful therapeutically for diabetic nephropathy [168]. P2X7R activation contributes to the high prevalence of kidney disease found in diabetics [160].

## Diseases of the lower urinary tract

Localised inhibition of P2X7R at the spinal cord inflammatory injury site in a rat model reduced microglia numbers and improved neurogenic bladder dysfunction [169]. P2X7R, expressed by macrophages and neutrophils in the bladder submucosa, are increased in cyclophosphamide-induced haemorrhagic cystitis in mice and treatment with A-438079 or genetic ablation of this receptor reduced the tissue levels of IL-1β and TNF-α and reduced nociceptive behaviour [170]. It was shown that 7 days after unilateral ureteral obstruction in WT mice there was increased expression of P2X7R associated with inflammation and fibrogenic responses in the cortex [171]. It was suggested that there is a potential role for P2X7R antagonists to prevent renal interstitial fibrosis [172].

## Diseases of the liver

In mouse models of acetaminophen (APAP)-induced inflammation, liver injury after overdose involved P2X7R activation [173] resulting in hepatic caspase-1 and migration of neutrophils into the liver, suggesting that ATP may play a pivotal role in the development of inflammasomes after APAP overdose [174]. A-438079 was reported to be protective against APAP-induced liver injury, by an effect on metabolic activation and cell death pathways, rather than on involvement of inflammasomes [175]. Blockade of the P2X7R-NLRP3 inflammasome axis is a potential therapeutic target for liver fibrosis [176]. In mice with induced autoimmune hepatitis, activation of P2X7R on natural killer T cells was shown to inhibit naive but stimulate activated cells, resulting in suppression or stimulation of the autoimmune hepatitis [177]. P2X7R-mediated responses participate in infection of human hepatocytes by hepatitis delta virus and hepatitis B virus. P2X7R mediate leptin-induced GLUT4 function in mice stellate cells in non-alcoholic steatohepatitis [178]. P2X7R may be a major component of the purinergic signalling complex in hepatitis C virus-induced liver pathogenesis [34].

## Skin diseases

A valuable review discusses the involvement of P2X7R in skin biology and their role in inflammatory skin disorders, including irritant and chronic dermatitis, psoriasis, cancer, graft-versus-host disease as well as wound healing and transplantation [179]. Inflammation of the skin is associated with release of ATP, increase in expression of purinoceptors, particularly the P2X7R, and subsequent release of proinflammatory cytokines from immune cells [180]. P2X7R antagonists reduce skin inflammation. In psoriasis, high P2X7R expression, confined to the cell membrane of the basal layer of the epidermis, plays a role in shaping the inflammatory microenvironment [181], leading to differentiation of Th17 lymphocytes, which are involved in the pathogenesis and potential treatment of psoriasis [182]. Systemic sclerosis fibroblasts express mRNA for several P2R subtypes, including P2X7R, suggesting a potential therapeutic role for P2X7R antagonists in systemic sclerosis patients. In the initial phase of wound healing, there is acute inflammation. P2X7R on immune cells also mediate killing of intracellular pathogens by inducing apoptosis of host macrophages, chemo-attraction and cell adhesion. P2X7R immunoreactivity was associated with hyperkeratotic areas of the stratum corneum and in nuclei of koilocytes in the suprabasal layers of warts [183]. The nuclei that were positive for P2X7R were shrunken, showing much more intense P2X7R staining. The presence of P2X7R in the nucleus of human papillomavirus-infected cells indicates disruption of the cellular machinery. P2X7R agonists may be used to trigger apoptosis in these virally infected cells. Sensitisation to contact allergens requires activation of the immune system by endogenous danger signals. Mice lacking P2X7R are resistant to contact hypersensitivity. P2X7R-deficient dendritic cells fail to induce sensitisation to contact allergens and do not release IL-1β in response to ATP, suggesting that P2X7R are crucial for extracellular ATP release in skin in response to contact allergens [184]. Interference with P2X7R signalling may be a promising strategy for the prevention of allergic contact dermatitis. There is a pathogenic role for keratinocyte-derived ATP in irritant dermatitis. The necrosis produced by the chemical irritant croton oil was prevented in mice by pre-treatment with A-438079 [185].

## Musculoskeletal diseases

P2X7R proteins were upregulated on dystrophic myoblasts of *mdx* mice (a mouse model of Duchenne muscular dystrophy) and it was suggested that antagonists to these receptors may be of potential therapeutic benefit [186] as treatment with BBG or oxATP slowed the progression of disease in the mouse model [187]. The P2X7R has received the most attention in relation to the treatment of osteoporosis [188]. P2X7R plays an important role in both cortical and cancellous bone mass augmentation. They were shown to mediate stimulation of periosteal and cancellous bone formation and inhibition of cancellous bone resorption during growth [188]. Single nucleotide polymorphisms (SNPs) of the P2X7 gene are associated with fracture risk, decrease in bone mineral density and osteoporosis [189]. P2X7R are also involved in the chain of events leading to the formation of human osteoclasts, and P2X7R antagonists may have therapeutic roles in bone diseases with an increase in osteoclast number, such as Paget’s disease [190]. P2X7R antagonists may be useful for the management of osteoporosis and with disorders of remodelling, where there is reduced bone mass [191]. Different polymorphic variants of the P2X7R are associated with high or reduced periprosthetic osteolysis in the long-term complication of total hip arthroplasty due to osteoarthritis [192]. Cross-talk between P2X7R and Wnt/β-catenin pathways may modulate osteoblast activity in response to mechanical loading [193]. Treatment with AZD9056, a selective P2X7R antagonist, produced pain-relieving and anti-inflammatory effects in rats with osteoarthritis [194]. ATP, via P2X7R, induced higher levels of IL-1β in blood samples from rheumatoid arthritis (RA) patients compared to controls. It was suggested that mononuclear cells from these patients were more sensitive to ATP stimulation, perhaps due to genetic polymorphism in the P2X7R gene [195]. P2X7R play a role in the pathogenesis of RA and systemic lupus erythematosus. Human rheumatoid synoviocytes express P2X7R mRNA and protein. Animal models of arthritis have provided evidence for an in vivo role for the P2X7R in the progression of inflammatory disease. In P2X7R KO mice, there was a reduced incidence and severity of anti-collagen-induced arthritis symptoms; therefore, targeting the P2X7R may be a promising treatment for RA [196]. Block of P2X7R signalling in the collagen-induced arthritis animal model of RA inhibited peripheral inflammatory tissue destruction. P2X7R antagonists have been explored for the treatment of inflammatory pain in joints [197]. Charcot-Marie-Tooth 1A is a demyelinating hereditary neuropathy and A-438079 improved the clinical phenotype of the disease in a rat model and was recommended for its treatment [198]. A review was published about P2X7R antagonists in rodent models of musculoskeletal and other disorders [199].

## P2X7R and diabetes

Type 1 and type 2 diabetes are inflammatory diseases. P2X7R KO in mice prevents streptozotocin (STZ)-induced type 1 diabetes and the levels of proinflammatory mediators (IL-1β, interferon-γ and nitric oxide) did not increase [200]. P2X7R-pannexin 1 channels impair bone mechanosignalling in osteocytes associated with type 1 diabetes that affects osteoblast function and maintenance of bone health [201]. In STZ-induced diabetic animals, P2X7R located on glucagon-containing α cells in pancreatic islets increase and they migrate centrally to take the place of the missing insulin-containing β cells [202]. OxATP in mice has been proposed as a therapeutic tool to cause immunosuppression and tolerance induction in pancreatic islet transplantation [203, 204]. LncRNA NONRATT021972 siRNA decreases the expression of P2X7 mRNA and protein in DRG, thereby reducing mechanical and thermal hyperalgesia in type 2 diabetic rats [205]. ATP concentrations and P2X7R expression were increased in glial cells in rats with painful diabetic neuropathy [206]. Diabetic sympathetic neuropathy in type 2 diabetic rats was improved by reducing the expression of P2X7R with lncRNAuc.48+ siRNA in superior cervical ganglia [207]. Different P2X7R polymorphisms are associated with pain sensitivity for diabetic neuropathic pain patients, such that patients with the gain-of-function SNP report more severe pain, whereas those with a loss-of-function SNP report less pain when the pain was tested and scored [208]. P2X7R antagonists may be a useful coadjuvant treatment to delay the progression of diabetic nephropathy [209].

## Conclusions

It is clear from the recent literature that P2X7R antagonists are potential effective therapeutic agents for the treatment of inflammatory diseases and cancer. There is a problem, however, concerning the many polymorphic forms of the human P2X7R, as care must be taken in identifying those patients that would most benefit by the available antagonists, as different antagonists are not always effective agents at some of the polymorphic types (see [210]). There is an explosion of interest in developing centrally penetrant P2X7R antagonists and their therapeutic explorations in clinical trials [211–213]. The relation between inflammation elicited by P2X7R activation and the immune system needs further exploration. Apart from the potential of the therapeutic use of P2X7R antagonists for CNS disorders (including neurodegenerative diseases, brain injury, psychiatric diseases, epilepsy and neuropathic pain), there is increasing recognition of their use for the treatment of diseases of the heart, lung, gut, kidney, liver and bladder. The reasons for dual roles of P2X7R mediating cell proliferation and apoptotic cell death need further investigation of the mechanisms involved.

The purinergic signalling field is now well established and much known about the physiological roles played by this system. The emphasis now is on the pathophysiology and therapeutic potential of purinergic signalling (see [214, 215]). For example, clopidogrel (trade name Plavix) and ticagrelor (trade name Brilique) are P2Y_12_ receptor antagonists that inhibit platelet aggregation and are widely used for the treatment of thrombosis and stroke (see [216]).

Characterisation of the P2Y class (see [217]) made it possible to identify specific agonists of the P2Y_2_ receptor, which evokes mucus secretion. A new, long-lasting agonist, diquafasol (Diquas), has been developed by Inspire Pharmaceuticals [218]. Diquas was launched by Santen in Japan in 2010, with Inspire Pharmaceuticals.

Through the cloning of the P2X3 ion channel receptor located on nociceptive sensory nerves [219] and later the discovery of the involvement of these channels in signalling pain (see [220]), a new target for pain relief is receiving attention from the pharmaceutical industry. Roche and, more recently, Afferent Pharmaceuticals have developed AF-219, a specific P2X3 antagonist, which is a promising new analgesic, orally bioavailable and stable in vivo [221]. AF-219 is currently in phase 2 clinical trials for the treatment of three painful disorders: osteoarthritis; interstitial cystitis/bladder pain syndrome and idiopathic chronic cough. Four phase 1 studies of AF-219 have demonstrated that the compound is safe and well tolerated and the phase 2 clinical trials were completed earlier this year [222]. Antagonism of P2X3 receptors on primary afferent neurons is a novel analgesic approach that has been pursued by leading pharmaceutical companies for the last 15 years. Over 600 patents are currently held which relate to the P2X3 receptor and pain (https://patentscope.wipo.int/search/en/result.jsf). There are currently investigations into the potential therapeutic use of purinergic compounds for osteoporosis, irritable bowel syndrome, atherosclerosis, kidney failure and cancer.

Since P2X7R-mediated inflammation is associated with a wide range of diseases, medicinal chemists in research institutes, universities and drug companies are optimistic that P2X7R antagonists are promising tools for the treatment of inflammatory diseases and more than 70 patents have been filed in the last few years by Glaxo, AstraZeneca, Roche, Janssen and Abbott, to name a few. For instance, Abbot filed a patent from the use of A-438079 in disease models of affective disorders and status epilepticus, while Abbott filed for triazole-based compounds for the CNS disorders and as a therapeutic strategy to treat spinal cord injury and the University Hospitals of Cleveland claimed in vitro and in vivo effects of P2X7R antagonists in epithelial cancer and papilloma (see [213]).

As mentioned above, a major challenge is to understand which polymorphic variations of the P2X7R have relevance to which disease and to fit patients with different polymorphisms to the P2X7R antagonist that would have the greatest efficacy for that variant. For example, the responses of human leukocytes to GSK1370319A were significantly altered, directly related to the SNP genotype, there being a 6.7-fold difference in the inhibition of ATP-stimulated IL-1β release by GSK1370319A between individuals with the homozygous gain- and loss-of-function genotypes [223]. A correlation was found between some gain-of-function and loss-of-function P2X7R SNPs, transfected into HEK-293 cells, and the expression of the channel protein. A change in both channel and pore function was described for the mutant P2X7R in parallel to the protein levels, although the agonist and antagonist sensitivity was not altered. The presence of the gain-of-function SNP (rs208294 (His155Tyr) and rs1718119 (Ala348Thr)) in female patients with diabetic peripheral neuropathic pain was associated with higher pain intensity scores [208]. Recently, mutations and molecular modelling studies have identified an allosteric binding site forming at the subunit interface at the apex of the receptor, distinct from the ATP-binding pocket, that regulates access of antagonists, such as AZ10606120, to the allosteric site. This allosteric pocket may provide novel targets for P2X7R drug development [224, 225], although the effect of polymorphic variations on the allosteric site has not been elucidated yet.

Also, more needs to be discovered about the relation of inflammatory diseases to the immune system where lymphocytes, macrophages, monocytes, neutrophils, basophils, dendritic cells, eosinophils and mast cells all express P2X7R (see [6]).

It seems likely that a breakthrough will occur about the use of P2X7R antagonists for a variety of diseases in the future, including, in particular, neurodegenerative diseases and cancer and the description of the crystal structure of mammalian P2X7R can only help in this endeavour [225, 226].

In addition to the therapeutic potential of P2X7R antagonists, P2X3R antagonists look extremely promising for the treatment of chronic cough, visceral pain, bladder disorders and hypertension. Exploration of the use of purinergic drugs for the treatment of obesity is also well worth pursuing.
